# Supplementary material for: Development of the MSKP index: Risk model of musculoskeletal pain in Colombian adolescents
Source: PLoS One. 2025 Aug 26;20(8):e0330500. doi: 10.1371/journal.pone.0330500 (PMC12380312; doi:10.1371/journal.pone.0330500)
Supplement: S1 Questionnaire — Original applied questionnaire, in spanish. (PDF) [file pone.0330500.s001.pdf]

30 minutes

Exposición a pantallas y comportamiento sedentario en adolescentes

* Required

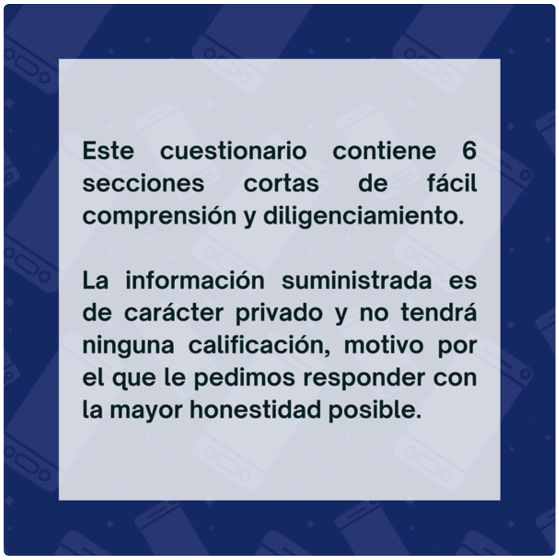

1

Escriba el código asignado *

SECCIÓN 1: INFORMACIÓN SOCIODEMOGRÁFICA

2

¿Cuál es su género? *

- Femenino
- Masculino
- Otro

3

Si su respuesta fue otro, ¿cuál?

4

¿Qué edad tiene? *

- 10 años
- 11 años
- 12 años
- 13 años
- 14 años
- 15 años
- 16 años
- 17 años
- 18 años

5

¿Usted ha sido diagnosticado por un médico con alguna discapacidad cognitiva o enfermedad importante que requiera tratamiento médico permanente? *

- ◯ Si
- ◯ No

6

Si su respuesta fue si, ¿cuál?

7

¿Con quién vive? Puede seleccionar más de una respuesta *

- ☐ Papá y/o mamá
- ☐ Hermano(s) y/o hermana(s)
- ☐ Abuelo(s) y/o abuela(s)
- ☐ Tío(s) y/o tía(s)
- ☐ Primo(s) y/o prima(s)
- ☐ Cuñado(s) y/o cuñada(s)
- ☐ Sobrino(s) y/o sobrina(s)
- ☐ Vive con una(s) persona(s) que no pertenecen a su familia

8

La zona en que usted vive es: *

- ◯ Rural
- ◯ Urbana

9

Su vivienda es: *

- Casa
- Apartamento

10

En su casa o apartamento, ¿qué actividades suele realizar? Puede seleccionar más de una respuesta *

- Tareas de limpieza del hogar (barrer o trapear)
- Tareas de lavado, tendido o planchado de ropa
- Tareas de cocina (preparar los alimentos o lavar los platos)
- Las compras en supermercado o plaza de mercado
- Tender la cama o arreglo del cuarto
- Paseo, cuidado y alimentación de mascota(s)
- Labores del campo (arreo de ganado, siembra, laboreo, abonado, riego o poda del campo)
- Cuidado de familiares u otras personas (asistiendo sus funciones en el hogar a personas mayores o acompañamiento de menores)

11

¿En qué colegio estudia? *

- Instituto Técnico Comercial José de San Martín sede Principal
- Instituto Técnico Comercial José de San Martín sede Camilo Torres
- Institución Educativa Diego Gómez de Mena sede Principal
- Institución Educativa Diego Gómez de Mena sede Antonio Nariño

12

¿Qué grado cursa? *

- Quinto
- Sexto
- Séptimo
- Octavo
- Noveno
- Décimo
- Once

13

Normalmente, ¿usted se desplaza de la casa hacia el colegio y del colegio a su casa a través de qué medio? *

- Ruta escolar
- Transporte público
- Carro o moto familiar o de otra persona
- Bicicleta
- Caminando
- Otro

14

Si su respuesta fue otro, ¿cuál?

SECCIÓN 2: EXPOSICIÓN Y DEPENDENCIA A LAS PANTALLAS DE DISPOSITIVOS MÓVILES

15

¿Usted tiene o cuenta con acceso a un celular? *

- Si
- No

16

¿Usted tiene acceso a una red WIFI en su celular en casa o el colegio? *

- Si
- No

17

¿Usted cuenta con un plan o paquete de datos móviles en su celular? *

- Si
- No

18

De lunes a viernes, normalmente ¿cuántas horas al día pasa en su celular en aplicaciones de mensajería móvil como WhatsApp, redes sociales como Instagram, Facebook, Twitter, Tik Tok, en aplicaciones de entretenimiento y juegos, o realizando labores del colegio como consultas en internet de tareas, preparación de pruebas o demás actividades que suele realizar? *

- Ninguna
- Menos de 2 horas al día
- Entre 2 y 4 horas al día
- Más de 4 horas al día

19

Los fines de semana, días festivos, vacaciones y días en que no asiste al colegio, normalmente ¿cuántas horas al día pasa en su celular en aplicaciones de mensajería móvil como WhatsApp, redes sociales como Instagram, Facebook, Twitter, Tik Tok, en aplicaciones de entretenimiento y juegos, o realizando labores del colegio como consultas en internet de tareas, preparación de pruebas o demás actividades que suele realizar? *

- Ninguna
- Menos de 2 horas
- Entre 2 y 4 horas al día
- Más de 4 horas al día

20

Indique con qué frecuencia realiza las afirmaciones que aparecen a continuación tomando como criterio la siguiente escala: (0) Nunca (1) Rara vez (2) A veces (3) Con frecuencia (4) Muchas veces *

	(0) Nunca	(1) Rara vez	(2) A veces	(3) Con frecuencia	(4) Muchas veces
Me han llamado la atención o me han hecho alguna advertencia por utilizar mucho el celular	◯	◯	◯	◯	◯
Me he puesto un límite de uso y no lo he podido cumplir	◯	◯	◯	◯	◯
He discutido con algún familiar por el gasto económico que hago del celular	◯	◯	◯	◯	◯
Dedico más tiempo del que quisiera a usar el celular	◯	◯	◯	◯	◯
Me he pasado (me he excedido) con el uso del celular	◯	◯	◯	◯	◯
Me he acostado más tarde o he dormido menos por estar utilizando el celular	◯	◯	◯	◯	◯
Gasto más dinero con el celular del que me había previsto	◯	◯	◯	◯	◯
Cuando me aburro, utilizo el celular	◯	◯	◯	◯	◯
Utilizo el celular en situaciones que, aunque no son peligrosas, no es correcto hacerlo (comiendo, mientras otras personas me hablan, etc.)	◯	◯	◯	◯	◯
Me han reñido (regañado) por el gasto económico del celular	◯	◯	◯	◯	◯

21

Indique en qué medida está de acuerdo o en desacuerdo con las afirmaciones que se presentan a continuación, siendo: (0) Totalmente en desacuerdo (1) Un poco en desacuerdo (2) Neutral (3) Un poco de acuerdo (4) Totalmente de acuerdo *

	Totalmente en desacuerdo	Un poco en desacuerdo	Neutral	Un poco de acuerdo	Totalmente de acuerdo
Cuando llevo un tiempo sin utilizar el celular, siento la necesidad de usarlo (llamar a alguien, enviar un SMS o un WhatsApp, etc.)	◯	◯	◯	◯	◯
Últimamente utilizo mucho más el celular	◯	◯	◯	◯	◯
Si se me estropeara (dañara) el celular durante un periodo largo de tiempo y tardarán en arreglarlo, me encontraría mal	◯	◯	◯	◯	◯
Cada vez necesito utilizar el celular con más frecuencia	◯	◯	◯	◯	◯
Si no tengo el celular me encuentro mal	◯	◯	◯	◯	◯
Cuando tengo el celular a la mano, no puedo dejar de utilizarlo	◯	◯	◯	◯	◯
Nada más levantarme lo primero que hago es ver si me ha llamado alguien al celular, si me han mandado un mensaje, un WhatsApp, etc.	◯	◯	◯	◯	◯
Cuando me siento solo, le hago una llamada a alguien, le envío un mensaje o un WhatsApp, etc.	◯	◯	◯	◯	◯
Gasto más dinero con el celular ahora que al principio	◯	◯	◯	◯	◯
Ahora mismo agarraría el celular y enviaría un mensaje, o [illegible]	◯	◯	◯	◯	◯

mensaje, o haría una llamada					
No es suficiente para mí usar el celular como antes, necesito usarlo cada vez más	○	○	○	○	○
No creo que pudiera aguantar una semana sin celular	○	○	○	○	○

SECCIÓN 3: EXPOSICIÓN A PANTALLAS DE OTROS DISPOSITIVOS ELECTRÓNICOS

22

¿Usted tiene en casa o cuenta con acceso a uno de los siguientes dispositivos electrónicos? Puede seleccionar más de una respuesta *

- ☐ Televisor
- ☐ Computador
- ☐ Tableta digital
- ☐ PlayStation
- ☐ Xbox
- ☐ Nintendo

23

De lunes a viernes, normalmente ¿cuántas horas al día utiliza alguno de los siguientes dispositivos electrónicos? *

	Ninguna	Menos de 2 horas al día	Entre 2 y 4 horas al día	Más de 4 horas al día
Televisor	○	○	○	○
Computador	○	○	○	○
Tableta digital	○	○	○	○
PlayStation	○	○	○	○
Xbox	○	○	○	○
Nintendo	○	○	○	○

24

Los fines de semana, días festivos, vacaciones y días en que no asiste al colegio, normalmente ¿cuántas horas al día utiliza alguno de los siguientes dispositivos electrónicos? *

	Ninguna	Menos de 2 horas al día	Entre 2 y 4 horas al día	Más de 4 horas al día
Televisor	◯	◯	◯	◯
Computador	◯	◯	◯	◯
Tableta digital	◯	◯	◯	◯
PlayStation	◯	◯	◯	◯
Xbox	◯	◯	◯	◯
Nintendo	◯	◯	◯	◯

SECCIÓN 4: CUESTIONARIO DE ACTIVIDAD FÍSICA PARA ADOLESCENTES PAQ-A

Queremos conocer cuál es su nivel de actividad física en los últimos 7 días (última semana). Esto incluye todas aquellas actividades como deportes, gimnasia o danza que hacen sudar o sentirse cansado, o juegos que hagan que acelere su respiración como saltar la cuerda, correr, etc. Recuerde que no hay preguntas buenas o malas y contesta las preguntas de las forma más honesta y sincera posible.

25

Actividad física durante su tiempo libre. ¿Ha hecho alguna de estas actividades en los últimos 7 días (última semana)? Si su respuesta es sí, ¿cuántas veces lo ha hecho? *

	No	1-2 días	3-4 días	5-6 días	7 o más días
Saltar la soga (cuerda o lazo)	◯	◯	◯	◯	◯
Patinar	◯	◯	◯	◯	◯
Juegos	◯	◯	◯	◯	◯
Montar en bicicleta	◯	◯	◯	◯	◯
Caminar (como ejercicio)	◯	◯	◯	◯	◯
Correr/footing	◯	◯	◯	◯	◯
Aeróbic/spinning	◯	◯	◯	◯	◯
Natación	◯	◯	◯	◯	◯
Bailar/danza	◯	◯	◯	◯	◯
Tenis	◯	◯	◯	◯	◯
Montar en skate	◯	◯	◯	◯	◯
Fútbol	◯	◯	◯	◯	◯
Voleibol	◯	◯	◯	◯	◯
Básquet	◯	◯	◯	◯	◯
Balonmano	◯	◯	◯	◯	◯
Atletismo	◯	◯	◯	◯	◯
Pesas	◯	◯	◯	◯	◯
Artes Marciales	◯	◯	◯	◯	◯
Otros	◯	◯	◯	◯	◯

26

En los últimos 7 días, durante las clases de educación física, ¿cuántas veces estuvo muy activo durante las clases: jugando intensamente, corriendo, saltando, haciendo lanzamientos? (Señale sólo una) *

- No hice/hago educación física
- Casi nunca
- Algunas veces
- A menudo
- Siempre

27

En los últimos 7 días, ¿qué hizo normalmente a la hora de la comida (antes y después de comer)? (Señale sólo una) *

- Estar sentado (hablar, leer, trabajo de clase)
- Pasear por los alrededores
- Correr o jugar un poco
- Correr y jugar bastante
- Correr y jugar intensamente todo el tiempo

28

En los últimos 7 días, inmediatamente después del colegio, ¿cuántos días jugó a algún juego, hizo deporte o bailes en los que estuviera muy activo? (Señale sólo uno) *

- Ninguna
- 1 vez en la última semana
- 2-3 veces en la última semana
- 4 veces en la última semana
- 5 veces o más en la última semana

29

En los últimos 7 días, ¿cuántos días a partir de las 6 pm y 10 pm hizo deportes, baile o juegos en los que estuviera muy activo? *

- Ninguna
- 1 vez en la última semana
- 2-3 veces en la última semana
- 4 veces en la última semana
- 5 veces o más en la última semana

30

El último fin de semana, ¿cuántas veces hizo deportes, baile o juegos en los que estuviera muy activo? (Señale sólo uno) *

- Ninguna
- 1 vez en la última semana
- 2-3 veces en la última semana
- 4 veces en la última semana
- 5 veces o más en la última semana

31

¿Cuál de las siguientes frases describe mejor su última semana? Lea las cinco alternativas antes de decidir cuál le describe mejor. (Señale sólo una) *

- Todo o la mayoría de mi tiempo libre lo dediqué a actividades que suponen poco esfuerzo físico
- Algunas veces (1 o 2 veces) hice actividades físicas en mi tiempo libre (por ejemplo: hace deportes, correr, nadar, montar en bicicleta, hacer aeróbicos)
- A menudo (3-4 veces a la semana) hice actividad física en mi tiempo libre
- Bastante a menudo (5-6 veces en la última semana) hice actividad física en mi tiempo libre
- Muy a menudo (7 o más veces a la semana) hice actividad física en mi tiempo libre)

32

Señale con qué frecuencia hizo actividad física para cada día de la semana (como hacer deporte, jugar, bailar, o cualquier otra actividad física) *

	Ninguna	Poca	Normal	Bastante	Mucha
Lunes	○	○	○	○	○
Martes	○	○	○	○	○
Miércoles	○	○	○	○	○
Jueves	○	○	○	○	○
Viernes	○	○	○	○	○
Sábado	○	○	○	○	○
Domingo	○	○	○	○	○

33

¿Estuvo enfermo en la última semana o algo impidió que hiciera normalmente actividades físicas? *

○ Si

○ No

SECCIÓN 5: CUESTIONARIO DE SÍNTOMAS MUSCULOESQUELÉTICOS

La imagen al costado derecho de su pantalla muestra cómo se ha dividido el cuerpo, usted debe seleccionar la zona sombreada en rojo, azul, morado o gris en que ha tenido dolor durante los últimos 6 meses marcando Si o No en la casilla correspondiente para cada pregunta.

34

¿Usted ha tenido dolor o molestia en el cuello (de color rojo) durante los últimos 6 meses?

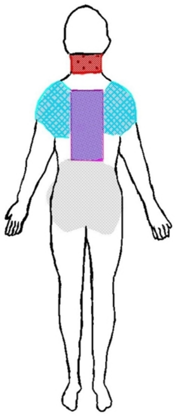

- ◯ Si
- ◯ No

35

¿En los últimos 6 meses, el dolor o molestia percibido en el cuello está acompañado de dolor de cabeza?

- ◯ Si
- ◯ No

36

¿Usted relaciona el origen del dolor o molestia, con el uso del celular o demás dispositivos tecnológicos?

- ◯ Si
- ◯ No

37

¿El dolor o molestia está presente únicamente mientras revisa su celular o está utilizando algún dispositivo tecnológico?

- Si
- No

38

¿El dolor o molestia está presente de forma permanente?

- Si
- No

39

¿Cuánto tiempo ha tenido dolor o molestia durante los últimos 6 meses en el cuello?

- 0 días
- 1 a 7 días
- 8 a 30 días
- Más de 30 días
- Todos los días

40

Usualmente, ¿cuánto tiempo dura la dolor en el cuello?

- Menos de 12 horas
- 12 a 24 horas
- 1 a 7 días
- Más de 1 semana

41

¿Cuál es la severidad del dolor en una escala de 0 a 10, teniendo en cuenta que 10 significa el dolor más fuerte que usted haya experimentado y cero nada de dolor?

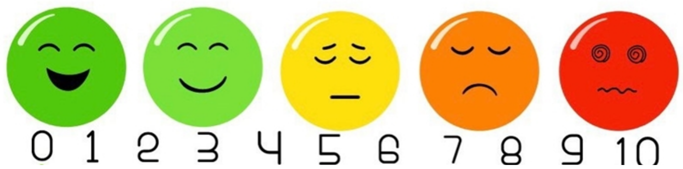

1	2	3	4	5	6	7	8	9	10

42

Durante los últimos 6 meses, ¿ha tenido que suspender o modificar sus actividades escolares debido al dolor, molestia o disconfort en el cuello?

- Si
- No

43

¿Cuánto tiempo estas molestias le han impedido realizar sus actividades escolares de la manera usual, en los últimos 6 meses?

- 0 días
- 1 a 7 días
- 1 a 4 semanas
- Más de 1 mes

44

Durante los últimos 6 meses, ¿ha visitado alguna vez a un médico, fisioterapeuta, quiropráctico u otra persona similar, o recibido otro tratamiento debido a la molestia en el cuello?

- Si
- No

45

Durante los últimos 6 meses, ¿ha tomado alguna vez medicamentos debido a la molestia en el cuello?

◯ Si

◯ No

46

¿Usted ha tenido molestia o dolor en el hombro (de color azul) durante los últimos 6 meses?

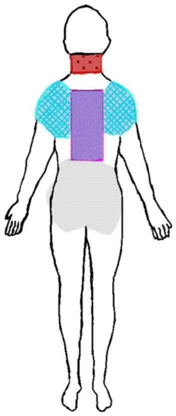

◯ Si

◯ No

47

¿Usted relaciona el origen del dolor o molestia, con el uso del celular o demás dispositivos tecnológicos?

◯ Si

◯ No

48

¿El dolor o molestia está presente únicamente mientras revisa su celular o está utilizando algún dispositivo tecnológico?

◯ Si

◯ No

49

¿El dolor o molestia está presente de forma permanente?

- Si
- No

50

¿Cuánto tiempo ha tenido dolor o molestia durante los últimos 6 meses en el hombro?

- 0 días
- 1 a 7 días
- 8 a 30 días
- Más de 30 días
- Todos los días

51

Usualmente, ¿cuánto tiempo dura la dolor en el hombro?

- Menos de 12 horas
- 12 a 24 horas
- 1 a 7 días
- Más de 1 semana

52

¿Cuál es la severidad del dolor en una escala de 0 a 10, teniendo en cuenta que 10 significa el dolor más fuerte que usted haya experimentado y cero nada de dolor?

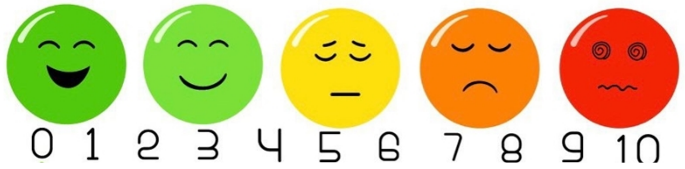

1	2	3	4	5	6	7	8	9	10

53

Durante los últimos 6 meses, ¿ha tenido que suspender o modificar sus actividades escolares debido al dolor, molestia o disconfort en el hombro?

- Si
- No

54

¿Cuánto tiempo estas molestias le han impedido realizar sus actividades escolares de la manera usual, en los últimos 6 meses?

- 0 días
- 1 a 7 días
- 1 a 4 semanas
- Más de 1 mes

55

Durante los últimos 6 meses, ¿ha visitado alguna vez a un médico, fisioterapeuta, quiropráctico u otra persona similar, o recibido otro tratamiento debido a la molestia en el hombro?

- Si
- No

56

Durante los últimos 6 meses, ¿ha tomado alguna vez medicamentos debido a la molestia en el hombro?

- Si
- No

57

¿Usted ha tenido molestia o dolor en la espalda alta (de color morado) durante los últimos 6 meses?

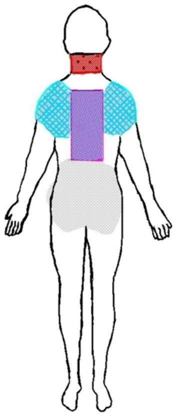

- Si
- No

58

¿Usted relaciona el origen del dolor o molestia, con el uso del celular o demás dispositivos tecnológicos?

- Si
- No

59

¿El dolor o molestia está presente únicamente mientras revisa su celular o está utilizando algún dispositivo tecnológico?

- Si
- No

60

¿El dolor o molestia está presente de forma permanente?

- Si
- No

61

¿Cuánto tiempo ha tenido dolor o molestia durante los últimos 6 meses en la espalda alta?

- 0 días
- 1 a 7 días
- 8 a 30 días
- Más de 30 días
- Todos los días

62

Usualmente, ¿cuánto tiempo dura la dolor en la espalda alta?

- Menos de 12 horas
- 12 a 24 horas
- 1 a 7 días
- Más de 1 semana

63

¿Cuál es la severidad del dolor en una escala de 0 a 10, teniendo en cuenta que 10 significa el dolor más fuerte que usted haya experimentado y cero nada de dolor?

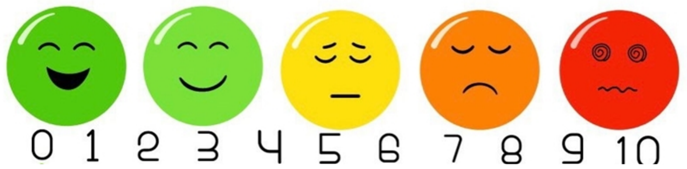

1	2	3	4	5	6	7	8	9	10

64

Durante los últimos 6 meses, ¿ha tenido que suspender o modificar sus actividades escolares debido al dolor o molestia en la espalda alta?

- ◯ Si
- ◯ No

65

¿Cuánto tiempo estas molestias le han impedido realizar sus actividades escolares de la manera usual, en los últimos 6 meses?

- ◯ 0 días
- ◯ 1 a 7 días
- ◯ 1 a 4 semanas
- ◯ Más de 1 mes

66

Durante los últimos 6 meses, ¿ha visitado alguna vez a un médico, fisioterapeuta, quiropráctico u otra persona similar, o recibido otro tratamiento debido a la molestia en la espalda alta?

- ◯ Si
- ◯ No

67

Durante los últimos 6 meses, ¿ha tomado alguna vez medicamentos debido a la molestia en la espalda alta?

- ◯ Si
- ◯ No

68

¿Usted ha tenido molestia o dolor en la espalda baja (de color gris) durante los últimos 6 meses?

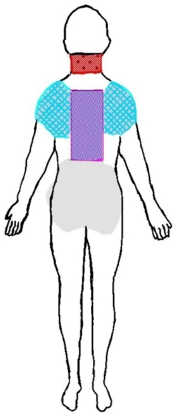

- Si
- No

69

¿Usted relaciona el origen del dolor o molestia, con el uso del celular o demás dispositivos tecnológicos?

- Si
- No

70

¿El dolor o molestia está presente únicamente mientras revisa su celular o está utilizando algún dispositivo tecnológico?

- Si
- No

71

¿El dolor o molestia está presente de forma permanente?

- Si
- No

72

¿Cuánto tiempo ha tenido dolor o molestia durante los últimos 6 meses en la espalda baja?

- 0 días
- 1 a 7 días
- 8 a 30 días
- Más de 30 días
- Todos los días

73

Usualmente, ¿cuánto tiempo dura la dolor en la espalda baja?

- Menos de 12 horas
- 12 a 24 horas
- 1 a 7 días
- Más de una semana

74

¿Cuál es la severidad del dolor en una escala de 0 a 10, teniendo en cuenta que 10 significa el dolor más fuerte que usted haya experimentado y cero nada de dolor?

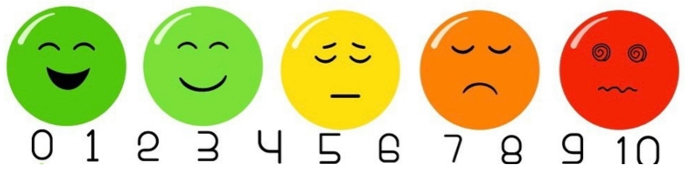

1	2	3	4	5	6	7	8	9	10

75

Durante los últimos 6 meses, ¿ha tenido que suspender o modificar sus actividades escolares debido al dolor, molestia o disconfort en la espalda baja?

- Si
- No

76

¿Cuánto tiempo estas molestias le han impedido realizar sus actividades escolares de la manera usual, en los últimos 6 meses?

- 0 días
- 1 a 7 días
- 1 a 4 semanas
- Más de 1 mes

77

Durante los últimos 6 meses, ¿ha visitado alguna vez a un médico, fisioterapeuta, quiropráctico u otra persona similar, o recibido otro tratamiento debido a la molestia en la espalda baja?

- Si
- No

78

Durante los últimos 6 meses, ¿ha tomado alguna vez medicamentos debido a la molestia en la espalda baja?

- Si
- No

SECCIÓN 6: ESCALA DE CALIDAD DEL SUEÑO PARA ESCOLARES

Responda las siguientes preguntas teniendo en cuenta los eventos ocurridos durante los últimos 7 días (última semana)

79

Usted duerme en: *

- Cama
- Hamaca
- Colchoneta en el suelo
- Mueble
- Otra

80

Si su respuesta fue otra, ¿cuál?

81

De lunes a viernes, normalmente ¿usted a qué hora se acuesta aproximadamente? Seleccione un rango de tiempo *

- Antes de las 7 p.m.
- 7 a 8 p.m.
- 8 a 9 p.m.
- 9 a 10 p.m.
- 10 a 11 p.m.
- 11 p.m a 12 a.m.
- Después de las 12 a.m.

82

De lunes a viernes, normalmente ¿usted a qué hora se despierta aproximadamente? Seleccione un rango de tiempo *

- Antes de las 3 a.m.
- 3 a 4 a.m.
- 4 a 5 a.m.
- 5 a 6 a.m.
- 6 a 7 a.m.
- 7 a 8 a.m.
- Después de las 8 a.m.

83

El fin de semana, normalmente ¿usted en qué hora se acuesta aproximadamente? Seleccione un rango de tiempo *

- Antes de las 7 p.m.
- 7 a 8 p.m.
- 8 a 9 p.m.
- 9 a 10 p.m.
- 10 a 11 p.m.
- 11 p.m. a 12 a.m.
- Después de las 12 a.m.

84

El fin de semana, normalmente ¿usted a qué hora se despierta aproximadamente? Seleccione un rango de tiempo *

- ○ Antes de las 3 a.m.
- ○ 3 a 4 a.m.
- ○ 4 a 5 a.m.
- ○ 5 a 6 a.m.
- ○ 6 a 7 a.m.
- ○ 7 a 8 a.m.
- ○ Después de las 8 a.m.

85

FACTOR 1: INICIO DEL SUEÑO. Con qué frecuencia en días, en la última semana, le sucedieron los siguientes eventos: *

	0 días	1-2 días	3-4 días	5-6 días	7 días
Se acostó pero no tenía sueño	○	○	○	○	○
Se sintió preocupado/a por no poder dormir	○	○	○	○	○
No durmió bien y no sabía por qué	○	○	○	○	○
No podía dormir a pesar de tener tiempo para hacerlo	○	○	○	○	○
No podía dormir a pesar de tener el espacio adecuado para hacerlo	○	○	○	○	○

86

FACTOR 2: PESADILLAS. Con qué frecuencia en días, en la última semana, le sucedieron los siguientes eventos: *

	0 días	1-2 días	3-4 días	5-6 días	7 días
Tuvo pesadillas	○	○	○	○	○
Despertó con miedo	○	○	○	○	○
Despertó sudando por algo que soñó	○	○	○	○	○
Soñó algo que le dio miedo	○	○	○	○	○

87

FACTOR 3: DESPERTARES NOCTURNOS. Con qué frecuencia en días, en la última semana, le sucedieron los siguientes eventos: *

	0 días	1-2 días	3-4 días	5-6 días	7 días
Despertó porque se atragantó	○	○	○	○	○
Le dijeron que despertó llorando pero usted no se acuerda	○	○	○	○	○
Despertó y sintió que no podía moverse	○	○	○	○	○
Le dijeron que despertó asustado/a y gritando pero usted no se acuerda	○	○	○	○	○
Roncó (se lo dijeron o lo sabe)	○	○	○	○	○

88

FACTOR 4: SOMNOLENCIA DIURNA. Con qué frecuencia en días, en la última semana, le sucedieron los siguientes eventos: *

	0 días	1-2 días	3-4 días	5-6 días	7 días
Tenía muchas ganas de dormir en el día	◯	◯	◯	◯	◯
Se durmió y soñó varias veces durante el día	◯	◯	◯	◯	◯
Con cerrar los ojos se dormía durante el día	◯	◯	◯	◯	◯
Se durmió viendo tele durante el día	◯	◯	◯	◯	◯

89

FACTOR 5: CANSANCIO Y DIFICULTADES PARA DESPERTAR. Con qué frecuencia en días, en la última semana, le sucedieron los siguientes eventos: *

	0 días	1-2 días	3-4 días	5-6 días	7 días
Despertó más cansado que cuando se acostó	◯	◯	◯	◯	◯
Sintió cansancio la mayor parte del día	◯	◯	◯	◯	◯
Fue difícil levantarse en la mañana	◯	◯	◯	◯	◯
Sintió necesidad de acostarse y levantarse más tarde que los demás	◯	◯	◯	◯	◯

90

FACTOR 6: SONAMBULISMO. Con qué frecuencia en días, en la última semana, le sucedieron los siguientes eventos: *

	0 días	1-2 días	3-4 días	5-6 días	7 días
Caminó dormido/a (se lo dijeron o lo sabe)	○	○	○	○	○
Se sentó o se paró dormido (se lo dijeron o lo sabe)	○	○	○	○	○
Habló dormido/a (se lo dijeron o lo sabe	○	○	○	○	○

This content is neither created nor endorsed by Microsoft. The data you submit will be sent to the form owner.

Microsoft Forms
