# Supplementary material for: Development of the MSKP index: Risk model of musculoskeletal pain in Colombian adolescents
Source: PLoS One. 2025 Aug 26;20(8):e0330500. doi: 10.1371/journal.pone.0330500 (PMC12380312; doi:10.1371/journal.pone.0330500)
Supplement: S2 Questionnaire — English translation of the applied questionnaire. (PDF) [file pone.0330500.s002.pdf]

Translation of the Questionnaire on Screen Exposure and Sedentary Behavior in Adolescents

This questionnaire consists of 6 short sections that are easy to understand and complete.
The information provided is confidential and will not be graded, so we kindly ask you to respond as honestly as possible.

1. **Write the assigned code: ___________**

SECTION 1: SOCIODEMOGRAPHIC INFORMATION

2. **What is your gender?**
    - Female
    - Male
    - Other
3. **If your answer was "Other," please specify:**
4. **How old are you?**
    - 10 years
    - 11 years
    - 12 years
    - 13 years
    - 14 years
    - 15 years
    - 16 years
    - 17 years
    - 18 years
5. **Have you been diagnosed by a doctor with any cognitive disability or significant illness requiring permanent medical treatment?**
    - Yes
    - No

6. **If your answer was "Yes," please specify:**
7. **Who do you live with? You may select more than one answer:**
    - Parent(s)
    - Sibling(s)
    - Grandparent(s)
    - Uncle(s)/Aunt(s)
    - Cousin(s)
    - Brother(s)-in-law/Sister(s)-in-law
    - Nephew(s)/Niece(s)
    - Person(s) outside of your family
8. **What type of area do you live in?**
    - Rural
    - Urban
9. **What type of housing do you live in?**
    - House
    - Apartment
10. **What household activities do you usually perform? You may select more than one answer:**
    - Cleaning tasks (sweeping or mopping)
    - Laundry-related tasks (washing, hanging, ironing clothes)
    - Cooking tasks (preparing meals or washing dishes)
    - Grocery shopping
    - Making the bed or tidying up the room
    - Walking, caring for, or feeding pets
    - Farm tasks (herding livestock, planting, fertilizing, watering, pruning)
    - Caring for relatives or other individuals (helping elderly people at home, or accompanying children)

11. **Which school do you attend?**

- Instituto Técnico Comercial José de San Martín, Principal Branch
- Instituto Técnico Comercial José de San Martín, Camilo Torres Branch
- Institución Educativa Diego Gómez de Mena, Principal Branch
- Institución Educativa Diego Gómez de Mena, Antonio Nariño Branch

12. **What grade are you in?**

- Fifth
- Sixth
- Seventh
- Eighth
- Ninth
- Tenth
- Eleventh

13. **How do you usually commute from home to school and back?**

- School bus
- Public transportation
- Family car or motorcycle
- Bicycle
- Walking
- Other

14. **If your answer was "Other," please specify:**

SECTION 2: SCREEN EXPOSURE AND MOBILE DEVICE DEPENDENCY

15. **Do you have access to a cell phone?**

- Yes
- No

16. **Do you have access to a Wi-Fi network on your cell phone at home or school?**

- Yes
- No

17. **Do you have a mobile data plan for your cell phone?**

- Yes
- No

18. **On weekdays, how many hours a day do you typically spend on your phone using mobile messaging apps (e.g., WhatsApp), social media (e.g., Instagram, Facebook, Twitter, TikTok), entertainment apps, or doing schoolwork (e.g., internet research, test preparation)?**

- None
- Less than 2 hours
- Between 2 and 4 hours
- More than 4 hours

19. **On weekends, holidays, or days when you do not attend school, how many hours a day do you typically spend on your phone using mobile messaging apps, social media, entertainment apps, or doing schoolwork?**

- None
- Less than 2 hours
- Between 2 and 4 hours
- More than 4 hours

20. **Indicate how often you experience the following situations based on the provided scale: (0) Never, (1) Rarely, (2) Sometimes, (3) Often, (4) Very Often**

- Someone has scolded or warned me about using my phone too much.
- I set a usage limit for myself but failed to follow it.
- I argued with a family member about the money I spend on my phone.
- I spend more time than I would like on my phone.
- I have exceeded my planned phone usage time.
- I have stayed up late or lost sleep because of using my phone.
- I spend more money on my phone than I had planned.
- When I'm bored, I use my phone.
- I use my phone in inappropriate situations (e.g., while eating, when talking to others).
- Someone has reprimanded me for the money I spend on my phone.

21. **Indicate the extent to which you agree or disagree with the following statements, where: (0) Strongly disagree, (1) Slightly disagree, (2) Neutral, (3) Slightly agree, (4) Strongly agree**

- When I go a while without using my phone, I feel the need to use it (call someone, send an SMS or WhatsApp, etc.).
- Lately, I've been using my phone much more.
- If my phone were to break for a long period of time and take a while to get fixed, I would feel bad.
- I need to use my phone more frequently each time.
- If I don't have my phone, I feel unwell.
- When I have my phone in hand, I can't stop using it.
- As soon as I wake up, the first thing I do is check if someone has called me, sent me a message, a WhatsApp, etc.
- When I feel lonely, I call someone, send them a message, or a WhatsApp, etc.
- I spend more money on my phone now than I did at the beginning.
- Right now, I would grab my phone and send a message or make a call.
- It's not enough for me to use my phone as I used to; I need to use it more and more.
- I don't think I could go a week without my phone.

- It’s not enough for me to use my phone as I used to; I need to use it more and more.
- I don’t think I could go a week without my phone.

SECTION 3: EXPOSURE TO OTHER ELECTRONIC DEVICES

22. **Do you have access to any of the following electronic devices at home? You may select more than one answer:**

- Television
- Computer
- Tablet
- PlayStation
- Xbox
- Nintendo

23. **On weekdays, how many hours a day do you typically use any of the following electronic devices? (0) None, (1) Less than 2 hours, (2) Between 2 and 4 hours, (3) More than 4 hours.**

- Television
- Computer
- Tablet
- PlayStation
- Xbox
- Nintendo

24. **On weekends, holidays, or days when you do not attend school, how many hours a day do you typically use any of the following electronic devices? (0) None, (1) Less than 2 hours, (2) Between 2 and 4 hours, (3) More than 4 hours.**

- Television

- Computer
- Tablet
- PlayStation
- Xbox
- Nintendo

SECTION 4: PHYSICAL ACTIVITY QUESTIONNAIRE FOR ADOLESCENTS (PAQ-A)

We would like to know your level of physical activity over the past 7 days (last week). This includes all activities such as sports, exercise, or dance that make you sweat or feel tired, or games that increase your breathing rate, such as jumping rope, running, etc. Remember, there are no right or wrong answers, so please answer the questions as honestly and sincerely as possible.

25. **Have you engaged in any of the following activities during your leisure time in the past 7 days (last week)? If your answer is yes, how many times have you done it? (0) No, (1) 1-2 days, (2) 3-4 days, (3) 5-6 days, (4) 7 days.**

- Jumping rope
- Skating
- Playing games
- Riding a bicycle
- Walking (as exercise)
- Running/jogging
- Aerobics/spinning
- Swimming
- Dancing
- Tennis
- Skateboarding
- Soccer

- Volleyball
- Basketball
- Handball
- Athletics
- Weightlifting
- Martial arts
- Others

26. **During the last 7 days, how often were you very active during physical education classes (e.g., playing intensely, running, jumping, throwing)? Select only one:**

- I did not/do not have physical education classes
- Almost never
- Sometimes
- Often
- Always

27. **During the last 7 days, what did you usually do during lunch breaks (before and after eating)? Select only one:**

- Sit (talk, read, do schoolwork)
- Walk around
- Run or play a little
- Run and play a lot
- Run and play intensely all the time

28. **During the last 7 days, immediately after school, how many days did you play a game, do sports, or dance, being very active? Select only one:**

- None
- 1 time in the past week
- 2-3 times in the past week

- 4 times in the past week
- 5 or more times in the past week

29. **During the last 7 days, between 6 pm and 10 pm, how many days did you do sports, dance, or games, being very active?**

- None
- 1 time in the past week
- 2-3 times in the past week
- 4 times in the past week
- 5 or more times in the past week

30. **During the last weekend, how many times did you do sports, dance, or games, being very active? Select only one:**

- None
- 1 time in the past weekend
- 2-3 times in the past weekend
- 4 times in the past weekend
- 5 or more times in the past weekend

31. **Which of the following statements best describes your last week? Read all five options before deciding which one applies best to you. Select only one:**

- I spent most or all of my free time doing activities that involve little physical effort.
- Sometimes (1 or 2 times) I did physical activities in my free time (e.g., playing sports, running, swimming, cycling, doing aerobics).
- Often (3-4 times a week) I did physical activity in my free time.
- Quite often (5-6 times in the last week) I did physical activity in my free time.
- Very often (7 or more times a week) I did physical activity in my free time.

32. **Indicate how often you did physical activity for each day of the week (e.g., playing sports, dancing, or any other physical activity): (0) Never, (1) Little, (2) Normal, (3) Quite a bit, (4) A lot.**

- Monday
- Tuesday
- Wednesday
- Thursday
- Friday
- Saturday
- Sunday

33. **Were you sick or did anything prevent you from engaging in physical activities as usual last week?**

- Yes
- No

SECTION 5: MUSCULOSKELETAL SYMPTOMS QUESTIONNAIRE

In the image on the right side of your screen, the body is divided into sections. Please indicate the shaded area (red, blue, purple, or gray) where you have experienced pain in the last 6 months by marking "Yes" or "No" for each question.

34. **Have you had pain or discomfort in your neck (red area) in the past 6 months?**

- Yes
- No

35. **In the last 6 months, has the neck pain or discomfort been accompanied by a headache?**

- Yes
- No

36. **Do you associate the origin of the pain or discomfort with the use of a cell phone or other technological devices?**

- Yes

- No

37. **Is the pain or discomfort present only when you check your cell phone or use any other technological device?**

- Yes
- No

38. **Is the pain or discomfort constant?**

- Yes
- No

39. **How long have you experienced neck pain or discomfort in the past 6 months?**

- 0 days
- 1-7 days
- 8-30 days
- More than 30 days
- Every day

40. **Typically, how long does the neck pain last?**

- Less than 12 hours
- 12-24 hours
- 1-7 days
- More than a week

41. **Rate the severity of your pain on a scale of 0 to 10, where 10 is the most severe pain you've experienced and 0 means no pain at all.**

42. **In the last 6 months, has neck pain caused you to stop or modify your school activities?**

- Yes
- No

43. **How long have these discomforts prevented you from performing your school activities normally in the past 6 months?**

- 0 days
- 1-7 days
- 1-4 weeks
- More than a month

44. **In the last 6 months, have you visited a doctor, physiotherapist, chiropractor, or similar person, or received any other treatment for neck discomfort?**

- Yes
- No

45. **In the last 6 months, have you taken any medication for neck discomfort?**

- Yes
- No

46. **Have you experienced pain or discomfort in your shoulder (blue area) in the past 6 months?**

- Yes
- No

47. **Do you associate the origin of the pain or discomfort in the shoulder with the use of a cell phone or other technological devices?**

- Yes
- No

48. **Is the pain or discomfort present only when you check your cell phone or use any other technological device?**

- Yes
- No

49. **Is the pain or discomfort in the shoulder constant?**

- Yes
- No

50. **How long have you experienced shoulder pain or discomfort in the past 6 months?**

- 0 days
- 1-7 days
- 8-30 days
- More than 30 days
- Every day

51. **Typically, how long does the shoulder pain last?**

- Less than 12 hours
- 12-24 hours
- 1-7 days
- More than a week

52. **Rate the severity of your shoulder pain on a scale of 0 to 10, where 10 is the most severe pain you've experienced and 0 means no pain at all.**

53. **In the last 6 months, has shoulder pain caused you to stop or modify your school activities?**

- Yes
- No

54. **How long have these discomforts prevented you from performing your school activities normally in the past 6 months?**

- 0 days
- 1-7 days
- 1-4 weeks
- More than a month

55. **In the last 6 months, have you visited a doctor, physiotherapist, chiropractor, or similar person, or received any other treatment for shoulder discomfort?**

- Yes

- No

56. **In the last 6 months, have you taken any medication for shoulder discomfort?**

- Yes
- No

57. **Have you experienced pain or discomfort in your upper back (purple area) in the past 6 months?**

- Yes
- No

58. **Do you associate the origin of the pain or discomfort in the upper back with the use of a cell phone or other technological devices?**

- Yes
- No

59. **Is the pain or discomfort in the upper back present only when you check your cell phone or use any other technological device?**

- Yes
- No

60. **Is the pain or discomfort in the upper back constant?**

- Yes
- No

61. **How long have you experienced upper back pain or discomfort in the past 6 months?**

- 0 days
- 1-7 days
- 8-30 days
- More than 30 days
- Every day

62. **Typically, how long does the upper back pain last?**

- Less than 12 hours
- 12-24 hours
- 1-7 days
- More than a week

63. **Rate the severity of your upper back pain on a scale of 0 to 10, where 10 is the most severe pain you've experienced and 0 means no pain at all.**
64. **In the last 6 months, has upper back pain caused you to stop or modify your school activities?**

- Yes
- No

65. **How long have these discomforts prevented you from performing your school activities normally in the past 6 months?**

- 0 days
- 1-7 days
- 1-4 weeks
- More than a month

66. **In the last 6 months, have you visited a doctor, physiotherapist, chiropractor, or similar person, or received any other treatment for upper back discomfort?**

- Yes
- No

67. **In the last 6 months, have you taken any medication for upper back discomfort?**

- Yes
- No

68. **Have you experienced pain or discomfort in your lower back (gray area) in the past 6 months?**

- Yes
- No

69. **Do you associate the origin of the pain or discomfort in the lower back with the use of a cell phone or other technological devices?**

- Yes
- No

70. **Is the pain or discomfort in the lower back present only when you check your cell phone or use any other technological device?**

- Yes
- No

71. **Is the pain or discomfort in the lower back constant?**

- Yes
- No

72. **How long have you experienced lower back pain or discomfort in the past 6 months?**

- 0 days
- 1-7 days
- 8-30 days
- More than 30 days
- Every day

73. **Typically, how long does the lower back pain last?**

- Less than 12 hours
- 12-24 hours
- 1-7 days
- More than a week

74. **Rate the severity of your lower back pain on a scale of 0 to 10, where 10 is the most severe pain you've experienced and 0 means no pain at all.**

75. **In the last 6 months, has lower back pain caused you to stop or modify your school activities?**

- Yes

- No

76. **How long have these discomforts prevented you from performing your school activities normally in the past 6 months?**

- 0 days
- 1-7 days
- 1-4 weeks
- More than a month

77. **In the last 6 months, have you visited a doctor, physiotherapist, chiropractor, or similar person, or received any other treatment for lower back discomfort?**

- Yes
- No

78. **In the last 6 months, have you taken any medication for lower back discomfort?**

- Yes
- No

SECTION 6: SLEEP QUALITY SCALE FOR SCHOOLCHILDREN

Answer the following questions based on the events that occurred over the past 7 days (last week).

79. **Where do you sleep?**

- Bed
- Hammock
- Mattress on the floor
- Couch
- Other

80. **If your answer was "Other," please specify:**

81. **On weekdays, what time do you usually go to bed? Select a time range:**

- Before 7:00 PM
- 7:00 - 8:00 PM
- 8:00 - 9:00 PM
- 9:00 - 10:00 PM
- 10:00 - 11:00 PM
- 11:00 PM - 12:00 AM
- After 12:00 AM

82. **On weekdays, what time do you usually wake up? Select a time range:**

- Before 3:00 AM
- 3:00 - 4:00 AM
- 4:00 - 5:00 AM
- 5:00 - 6:00 AM
- 6:00 - 7:00 AM
- 7:00 - 8:00 AM
- After 8:00 AM

83. **On weekends, what time do you usually go to bed? Select a time range:**

- Before 7:00 PM
- 7:00 - 8:00 PM
- 8:00 - 9:00 PM
- 9:00 - 10:00 PM
- 10:00 - 11:00 PM
- 11:00 PM - 12:00 AM
- After 12:00 AM

84. **On weekends, what time do you usually wake up? Select a time range:**

- Before 3:00 AM

- 3:00 - 4:00 AM
- 4:00 - 5:00 AM
- 5:00 - 6:00 AM
- 6:00 - 7:00 AM
- 7:00 - 8:00 AM
- After 8:00 AM

85. **FACTOR 1: SLEEP ONSET. How frequently have you experienced the following events in the last week? (0) 0 days, (1) 1-2 days, (2) 3-4 days, (3) 5-6 days, (4) 7 days.**

- Went to bed without feeling sleepy
- Felt worried about not being able to sleep
- Could not sleep well without knowing why
- Could not sleep despite having time to do so
- Could not sleep despite having an adequate space to do so

86. **FACTOR 2: NIGHTMARES. How frequently have you experienced the following events in the last week? (0) 0 days, (1) 1-2 days, (2) 3-4 days, (3) 5-6 days, (4) 7 days.**

- You had nightmares
- You woke up feeling scared
- You woke up sweating because of something you dreamed
- You dreamed of something that frightened you

87. **FACTOR 3: NIGHT AWAKENINGS. How frequently have you experienced the following events in the last week? (0) 0 days, (1) 1-2 days, (2) 3-4 days, (3) 5-6 days, (4) 7 days.**
    - You woke up because you were choking
    - You were told you woke up crying, but you don’t remember
    - You woke up and felt like you couldn’t move
    - You were told you woke up scared and screaming, but you don’t remember

- You snored (you were told or you know it)

88. **FACTOR 4: DAYTIME SLEEPINESS. How frequently have you experienced the following events in the last week? (0) 0 days, (1) 1-2 days, (2) 3-4 days, (3) 5-6 days, (4) 7 days.**

- You felt very sleepy during the day
- You fell asleep and dreamed several times during the day
- As soon as you closed your eyes, you would fall asleep during the day
- You fell asleep while watching TV during the day

89. **FACTOR 5: FATIGUE AND DIFFICULTY WAKING UP. How frequently have you experienced the following events in the last week? (0) 0 days, (1) 1-2 days, (2) 3-4 days, (3) 5-6 days, (4) 7 days.**

- You woke up feeling more tired than when you went to bed
- You felt tired most of the day
- It was difficult to get up in the morning
- You felt the need to go to bed and wake up later than others

90. **FACTOR 6: SLEEPWALKING. How frequently have you experienced the following events in the last week? (0) 0 days, (1) 1-2 days, (2) 3-4 days, (3) 5-6 days, (4) 7 days.**

- You walked while asleep (you were told or you know it)
- You sat or stood up while asleep (you were told or you know it)
- You talked while asleep (you were told or you know it)
