# Supplementary material for: Development of the MSKP index: Risk model of musculoskeletal pain in Colombian adolescents
Source: PLoS One. 2025 Aug 26;20(8):e0330500. doi: 10.1371/journal.pone.0330500 (PMC12380312; doi:10.1371/journal.pone.0330500)
Supplement: S3 Appendix — Contains the detail of the variables considered in the model (Table S3.1), and the correlation dendrogram (Figure S3.1). (PDF) [file pone.0330500.s003.pdf]

Appendix:

Table S3. 1. Complete list of the measured variables in the database, their meaning and their numerical operationalization. The variables are sorted alphabetically.

Code of variable	Name of variable	Operationalization
Activity PAQ-A	Physical Activity IPAQ†	1 (no activity) to 5 (frequent activity)
Activity_aerobics	Physical activity aerobics†	1 (no activity) to 5 (frequent activity)
Activity-after school	Physical activity after school†	1 (no activity) to 5 (frequent activity)
Activity athletism	Physical activity athletism†	1 (no activity) to 5 (frequent activity)
Activity-basket	Sport basketball †	1 (no activity) to 5 (frequent activity)
Activity_Dancing	Physical activity dancing†	1 (no activity) to 5 (frequent activity)
Activity_Dinner	Physical activity after dinner†	1 (no activity) to 5 (frequent activity)
Activity_evening	Physical activity in the evening†	1 (no activity) to 5 (frequent activity)
Activity_football	Sport Football†	1 (no activity) to 5 (frequent activity)
Activity_handball	Sport handball†	1 (no activity) to 5 (frequent activity)
Activity_illness past week	Illness during the past week	0=not/ 1=yes
Activity_jump-rope	Physical activity jump-rope†	1 (no activity) to 5 (frequent activity)
Activity_last week	Physical activity during last week	1 (no activity) to 5 (frequent activity)
Activity_martial arts	Sport martial arts†	1 (no activity) to 5 (frequent activity)
Activity_others	Another kind of Physical activity †	1 (no activity) to 5 (frequent activity)
Activity_playing	Physical activity playing†	1 (no activity) to 5 (frequent activity)
Activity_running	Sport Running†	1 (no activity) to 5 (frequent activity)
Activity_skateboard	Sport skateboard†	1 (no activity) to 5 (frequent activity)
Activity_skating	Sport skating†	1 (no activity) to 5 (frequent activity)
Activity_swimming	Sport Swimming†	1 (no activity) to 5 (frequent activity)
Activity tennis	Sport tennis†	1 (no activity) to 5 (frequent activity)
Activity volley	Sport volley†	1 (no activity) to 5 (frequent activity)
Activity_walking	Physical activity walking	1 (no activity) to 5 (frequent activity)
Activity_weigthlifting	Sports weightlifting†	1 (no activity) to 5 (frequent activity)
Age group 10-12	Age between 10 and 12 years	0=not/ 1=yes
Age group 13-15	Age between 13 and 15 years	0=not/ 1=yes
Age group 16-18	Age between 16 and 18 years	0=not/ 1=yes

Gender	Sex	0=male/ 1=female
Home agriculture	Agricultural activities at home	0=not/ 1=yes
Home area	Home is in a rural or urban area	0=rural/ 1=urban
Home kitchen	Kitchen cleaning at home	0=not/ 1=yes
Home Laundry	Laundry at home	0=not/ 1=yes
Home pets	In charge of pets are home	0=not/ 1=yes
Home room	Room cleaning at home	0=not/ 1=yes
home shopping	Home shopping	0=not/ 1=yes
Mobile data plan	Access to mobile data plan	0=not/ 1=yes
Mobile dependency	Mobile dependency test score	Total score of the Mobile dependence test: 0 (no dependency) to 74 (high dependency)
Mobile wifi	Access to a wifi network at home	0=not/ 1=yes
School area	Whether the school is in a rural or urban location	0=rural/ 1=urban
School_transport_0	Going to school by school provided transportation	0=not/ 1=yes
School_transport_1	Going to school by public transportation	0=not/ 1=yes
School_transport_2	Going to school by private transportation	0=not/ 1=yes
School_transport_3	Going to school by bike	0=not/ 1=yes
School_transport_4	Going to school by walking	0=not/ 1=yes
School_transport_5	Going to school by a different mean	0=not/ 1=yes
Sleep Factor_1	Sleep Factor 1 Difficulty initiating sleep	Scale from 0 (never) to 4 (frequent problem)
Sleep Factor_2	Sleep Factor 2 Nightmares	Scale from 0 (never) to 4 (frequent problem)
Sleep Factor_3	Sleep Factor 3 Night awakenings	Scale from 0 (never) to 4 (frequent problem)
Sleep Factor_4	Sleep Factor 4 Somnambulism	Scale from 0 (never) to 4 (frequent problem)
Sleep Factor_5	Sleep Factor 5 Tiredness and difficulty waking up	Scale from 0 (never) to 4 (frequent problem)
Sleep Factor_6	Sleep Factor 6 Daytime sleepiness	Scale from 0 (never) to 4 (frequent problem)

† These questions include Monday to Friday routine and weekends.

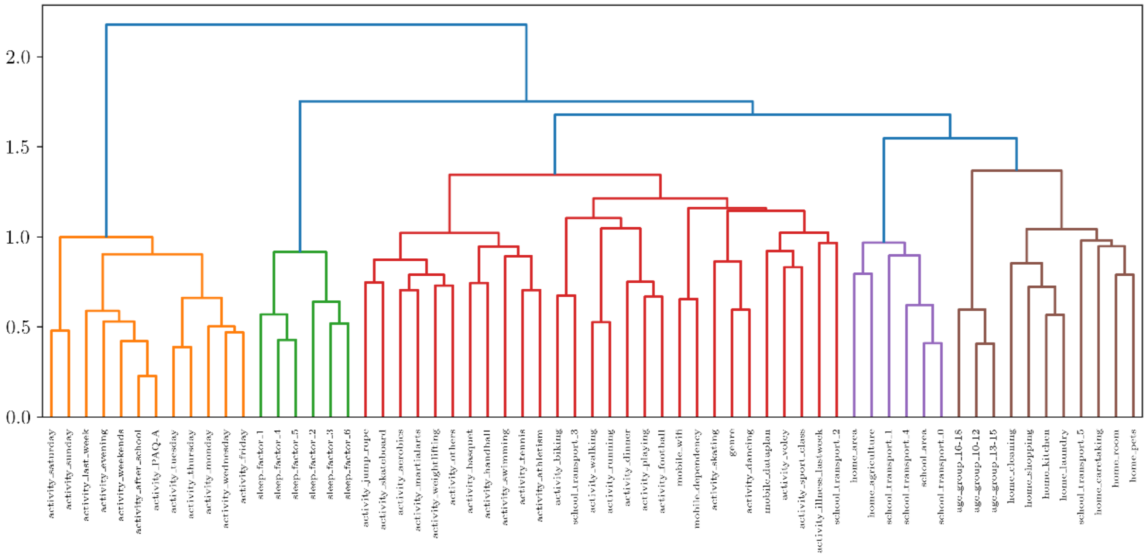

Figure S3. 1. Correlation hierarchy for all the measured variables in the dataset. Lower connections imply a stronger correlation between variables. The correlation is computed using the Spearman correlation.
