# Supplementary material for: Development of the MSKP index: Risk model of musculoskeletal pain in Colombian adolescents
Source: PLoS One. 2025 Aug 26;20(8):e0330500. doi: 10.1371/journal.pone.0330500 (PMC12380312; doi:10.1371/journal.pone.0330500)
Supplement: Attachment — Submitted filename: PLOS ONE.pdf [file pone.0330500.s004.pdf]

Authors and Editor, Dear

This manuscript, "MSKP INDEX: RISK MODEL OF MUSCULOSKELETAL PAIN IN A COLOMBIAN ADOLESCENT SAMPLE," presents an intriguing effort to develop a logistic regression-based risk model for musculoskeletal pain (MSKP) among Colombian adolescents. The study leverages a sizable sample (n=680) and addresses a regionally underexplored issue, proposing a novel MSKP index and risk scale with potential practical applications. The use of validated tools and advanced statistical methods (e.g., Lasso regularization, cross-validation) demonstrates methodological ambition. However, the manuscript requires significant revisions to meet the rigorous standards of a JCR-indexed journal like PLOS ONE. Key concerns include methodological ambiguities (e.g., MSKP index validation, feature selection details), statistical shortcomings (e.g., lack of overfitting checks, unadjusted odds ratios), and interpretive overreach (e.g., implied causality in a cross-sectional design). Below, I outline the main criticisms for each section, offering specific recommendations to enhance clarity, rigor, and impact. I recommend major revisions to address these issues, confident that the study's potential can be realized with careful refinement.

Title and Keywords

- Revise the title to eliminate redundancy (e.g., "Development of the MSKP Index: A Risk Model for Musculoskeletal Pain in Colombian Adolescents") and consider adding methodological specificity (e.g., "Logistic Regression Model").
- Replace "Physiotherapy" in keywords with "Logistic Regression" or "Risk Assessment" to align with the study's core methodology.

Abstract

- Specify the MSKP prevalence (e.g., "22.6% of participants reported MSKP") to enhance informativeness.
- Detail the methods by briefly mentioning feature selection (e.g., "using correlation analysis and Lasso regularization") and validation (e.g., "cross-validated on a test set").
- Trim the abstract to fit *PLOS ONE*'s 200–250-word limit while retaining essential details, ensuring conciseness without sacrificing clarity.

Introduction

- Expand the literature review to include a broader geographical and methodological scope (e.g., European studies like Hestbaek et al., 2006, *Spine*).
- Use cautious language to avoid unsupported causal claims (e.g., "may contribute to" instead of "trigger" for posture and pain).
- Substantiate the Latin American research gap with evidence (e.g., cite a review or meta-analysis).
- Articulate a specific hypothesis (e.g., "We hypothesized that mobile dependency and sleep quality would be significant predictors of MSKP") to provide a theoretical foundation.

Methods

- Address potential seasonal effects of data collection (September–October 2022) on physical activity or pain reporting.
- Recalculate sample size using a realistic prevalence estimate (e.g., 20–30% from prior studies) instead of the unjustified 50%, and report a power analysis.
- Acknowledge non-probabilistic sampling as a limitation and clarify vague exclusion criteria (e.g., specify types of "diagnosed disabilities").
- Validate the MSKP index cutoff (≥4) against clinical standards (e.g., pain duration, functional impairment) and justify pain intensity categories (1–3, 4–7, >7) with psychometric evidence.

- Provide reliability statistics for all instruments (e.g., IPAQ, sleep scale), not just TDM.
- Detail feature selection by specifying the correlation threshold and Lasso regularization parameters (e.g., lambda).
- Justify the cross-validation scheme (20x 3-fold) relative to sample size and clarify the standardization method (e.g., z-scoring).
- Adjust odds ratios (ORs) for multiple comparisons (e.g., Bonferroni) to reduce type I error risk.

Results

- Add confidence intervals or p-values to Table 1 for prevalence estimates and contextualize the mobile dependency score (e.g., clinical cutoff).
- Discuss the correlation dendrogram (Figure A.1) in the text to guide interpretation.
- Benchmark the Lasso model AUC (0.78) against simpler models (e.g., univariate regression) and quantify ROC curve variance (e.g., SD of AUC).
- Assess overfitting risk in the final model (e.g., via calibration plots or Brier score) and report sensitivity/specificity for each risk scale cutoff (Figure 4c).
- Test linearity assumptions for ORs (e.g., sleep_factor_5 OR = 7.89) and discuss coefficient instability indicated by wide CIs (e.g., [4.72–11.9]).

Discussion

- Use precise language (e.g., "associated with" instead of "correlated") to reflect logistic regression results accurately.
- Balance emphasis on mobile dependency (OR = 1.72) relative to stronger predictors like sleep factors, avoiding overhyping its role.
- Hypothesize mechanisms for football’s protective effect (OR = 0.66), such as aerobic fitness or coordination, to deepen interpretation.
- Engage critically with prior studies (e.g., explain differences from Thailand/Japan findings) rather than offering superficial comparisons.
- Benchmark the MSKP index against existing tools (e.g., Nordic Musculoskeletal Questionnaire) to substantiate novelty claims.

Conclusion

- Specify the MSKP index’s intended application (e.g., "for screening high-risk adolescents") rather than using vague terms like "promising."
- Avoid causal language (e.g., "contributing to this risk") given the cross-sectional design, emphasizing the need for longitudinal validation.

Limitations

- Acknowledge additional limitations: non-probabilistic sampling, lack of external validation, and potential overfitting risk.
- Clarify the ambiguous suggestion of "multiple regression models" (e.g., specify multinomial logistic regression or another approach).

Additional Sections

- Complete funding details per *PLOS ONE* guidelines (e.g., include grant amount for Project ID: ENF-59-2020).
- Resolve the placeholder Harvard Dataverse DOI ("XXX") with a functional link.
- Update references to include key predictive modeling studies (e.g., Hestbaek et al., 2006; Dunn et al., 2013) and address timeline issues (e.g., remove or justify 2024 citations post-dating 2022 data collection).
