# Supplementary material for: Development of the MSKP index: Risk model of musculoskeletal pain in Colombian adolescents
Source: PLoS One. 2025 Aug 26;20(8):e0330500. doi: 10.1371/journal.pone.0330500 (PMC12380312; doi:10.1371/journal.pone.0330500)
Supplement: Attachment — Submitted filename: RebutalLetter.pdf [file pone.0330500.s005.pdf]

Chía, Colombia. June 26th 2025

Editors of PLOS ONE

Rebutal Letter

PONE-D-24-55797

MSKP INDEX: RISK MODEL OF MUSCULOSKELETAL PAIN IN A COLOMBIAN ADOLESCENT SAMPLE

Dear Editors,

We would like to thank you and the reviewers of our manuscript for their insightful feedback. We have carefully addressed each of the reviewers' comments and are confident that the new version of the manuscript will fulfil the publication standards of the PLOS ONE journal.

In the following we respond to each of the reviewers' comments.

Reviewer 1:

Comment	Response
Revise the title to eliminate redundancy (e.g., "Development of the MSKP Index: A Risk Model for Musculoskeletal Pain in Colombian Adolescents") and consider adding methodological specificity (e.g., "Logistic Regression Model").	We have adapted the title of the manuscript to "Development of the MSKP Index: A Risk Model for Musculoskeletal Pain in Colombian Adolescents")
Replace "Physiotherapy" in keywords with "Logistic Regression" or "Risk Assessment" to align with the study's core methodology.	We will include the new keywords in the Manuscript as suggested by the reviewer
Specify the MSKP prevalence (e.g., "22.6% of participants reported MSKP") to enhance informativeness.	All suggestion made by the reviewer to the abstract have been incorporated in the new version of the manuscript. The abstract has been revised to meet the journal's suggested word limit, presenting the findings more concisely and clearly.
Detail the methods by briefly mentioning feature selection (e.g., "using correlation analysis	

and Lasso regularization") and validation (e.g., "cross-validated on a test set"	
Trim the abstract to fit PLOS ONE’s 200–250-word limit while retaining essential details, ensuring conciseness without sacrificing clarity.	
Expand the literature review to include a broader geographical and methodological scope (e.g., European studies like Hestbaek et al., 2006, Spine).	The introduction was reviewed and revised for clarity, the suggested references were incorporated, and the hypothesis was added.
Use cautious language to avoid unsupported causal claims (e.g., "may contribute to" instead of "trigger" for posture and pain).	
Substantiate the Latin American research gap with evidence (e.g., cite a review or meta-analysis).	
Articulate a specific hypothesis (e.g., "We hypothesized that mobile dependency and sleep quality would be significant predictors of MSKP") to provide a theoretical foundation.	
Address potential seasonal effects of data collection (September–October 2022) on physical activity or pain reporting.	We understand the reviewer’s concern. However, no seasonal effects on pain incidence are attributed to Colombia's location, as it is situated in a tropical zone where seasonal variations do not significantly influence environmental factors related to pain.
Recalculate sample size using a realistic prevalence estimate (e.g., 20–30% from prior studies) instead of the unjustified 50%, and report a power analysis.	We appreciate the comment of the reviewer. In the following we explain how we addressed the sample size determination.<br>In the Methods section, we stated that the sample size was determined utilizing Epidat 3.1. This calculation considered "a population size of 39,640 students, an anticipated proportion of 50%, a confidence level of 95%, and a precision of 5%". The use of an anticipated proportion of 50% is a standard practice in sample size calculation when the true prevalence of the condition of interest in the study population is unknown at the outset. This value is chosen because it maximizes the required sample size to estimate a proportion with given precision and confidence, thus ensuring a conservatively large sample size. This comment has been included in the updated version of the manuscript.<br>As for the suggested power analysis, our primary objective was to develop a logistic regression model to assess the risk of MSKP and identify associated factors. A formal *a priori* power analysis for multivariate models like logistic regression is complex, as it would need to calculate the study's ability to detect specific effect sizes (Odds Ratios) for each predictor variable, given their

	distributions and correlations. Instead of a detailed *a priori* power analysis for every predictor, the study evaluated the overall predictive capability of the constructed model with the achieved sample size. The results demonstrate that the final model had an Area Under the Curve (AUC) of 0.83 on the test set, which provides strong evidence of the model's significant capability to distinguish between positive and negative cases of MSKP using the observed data.<br>Furthermore, the study reports the Odds Ratios (ORs) and their 90% confidence intervals (90% CIs) for the 13 variables selected in the final model. These confidence intervals serve as a measure of the precision with which the effect of each relevant predictor variable is estimated. A narrower CI indicates a more precise estimate of the true effect, which is directly related to the statistical power for that specific variable given the sample size and data variability. The presentation of these CIs allows readers to evaluate if the study had sufficient precision to identify significant associations for the variables retained in the model.
Acknowledge non-probabilistic sampling as a limitation and clarify vague exclusion criteria (e.g., specify types of "diagnosed disabilities").	We have included the non-probabilistic sampling as a limitation. Also, we have elaborated on the exclusion criteria in the Participants paragraph.
Validate the MSKP index cutoff (≥4) against clinical standards (e.g., pain duration, functional impairment) and justify pain intensity categories (1–3, 4–7, >7) with psychometric evidence.	The proposed MSKP index assigns numerical scores to pain intensity in four regions, with a cutoff of ≥4 indicating significant pain severity, providing a quantitative measure of pain burden. In contrast, the Nordic Musculoskeletal Questionnaire primarily captures the presence or absence of pain across regions without a standardized score or severity thresholds, focusing on prevalence and distribution rather than intensity.
Provide reliability statistics for all instruments (e.g., IPAQ, sleep scale), not just TDM.	All references with the psychometric properties of the instruments used in the research project were included.
Detail feature selection by specifying the correlation threshold and Lasso regularization parameters (e.g., lambda).	We thank the reviewer for the comment. In the first feature selection step we used a correlation analysis, the primary goal of this step was to filter input variables based on correlation analysis to avoid collinearity, which can negatively affect the performance and stability of logistic regression models. The feature selection procedure was not carried out in terms of a correlation threshold; it involved both the correlation and the hierarchy dendrogram shown in Figure A.1. of the appendix instead. The dendrogram allows the identification of largely correlated feature groups, the correlation allows the determination of the largest correlations in each group and raise potential candidate features to be discarded. This process did not involve a formal or automated procedure and should be perceived as a correlation-driven feature screening.<br>In the second step, a formal Lasso regularization was performed. The lasso regularization parameter was among the optimized hyperparameters, the results shown in the paper correspond to a regularization parameter alpha = 10 (C=0.1, in scikit-learn). The Lasso regularization and the proposed cross-validation scheme clearly provide a robust statistical base to select the features in the final model.

Justify the cross-validation scheme (20x 3-fold) relative to sample size and clarify the standardization method (e.g., z-scoring).	The decision to implement a cross-validation scheme was made after our research team noticed significant variations in the results upon different definitions of the train-test sets. This is a common observation when dealing with relatively small samples. To mitigate these sample size effects, we decided to perform the cross-validation. The cross-validation offers in addition the advantage of providing a distribution for each of the feature coefficients. This distribution allows the confidence intervals and strengthens our analysis. In this sense, the cross-validation is used as a bootstrapping mechanism with a virtually infinite number of possibilities. The 20x3-fold scheme was selected to ensure that we obtain enough statistics while keeping the processing times within reasonable windows.
Adjust odds ratios (ORs) for multiple comparisons (e.g., Bonferroni) to reduce type I error risk.	We thank the reviewer for this important suggestion. We have corrected the confidence intervals in the odd ratios using an effective significance level $\alpha\_eff=\alpha/N$, where $\alpha=0.1$ is our required significance level (90% confidence interval), and $N=13$ is the number of features in our model. In the process of re-calculating the Odd Ratios we realized that in the previous manuscript we had unintentionally calculated the OR using the results of the first Lasso regression. In the updated version of the manuscript, we correctly use the results of the second regression step, using only a limited set of 13 features. As a result, we obtain much narrower confidence intervals, which evidence the robustness of our approach. The updated vales can be found in Table 2 of the Manuscript.
Add confidence intervals or p-values to Table 1 for prevalence estimates and contextualize the mobile dependency score (e.g., clinical cutoff).	These suggested changes have been included into Table 1 in the manuscript.
Discuss the correlation dendrogram (Figure A.1) in the text to guide interpretation.	The discussion of the dendrogram and the first feature selection process has been extended in the manuscript.
Benchmark the Lasso model AUC (0.78) against simpler models (e.g., univariate regression) and quantify ROC curve variance (e.g., SD of AUC).	The standard deviation of the AUC has been included in the figures and is also addressed in the manuscript text. The following Figure shows the AUC of the best univariate models for each of the selected features after the first feature selection step. As can be seen, none of the individual features performs as good as the collective Lasso model, the corresponding AUC for the Lasso model is shown in red. It should be noted, however, that the variable sleep_factor_5 alone has a large AUC of about 0.75. As discussed in the conclusions, this variable is the most important for the model and yields the largest ORs.

<table>
<tr><td></td><td>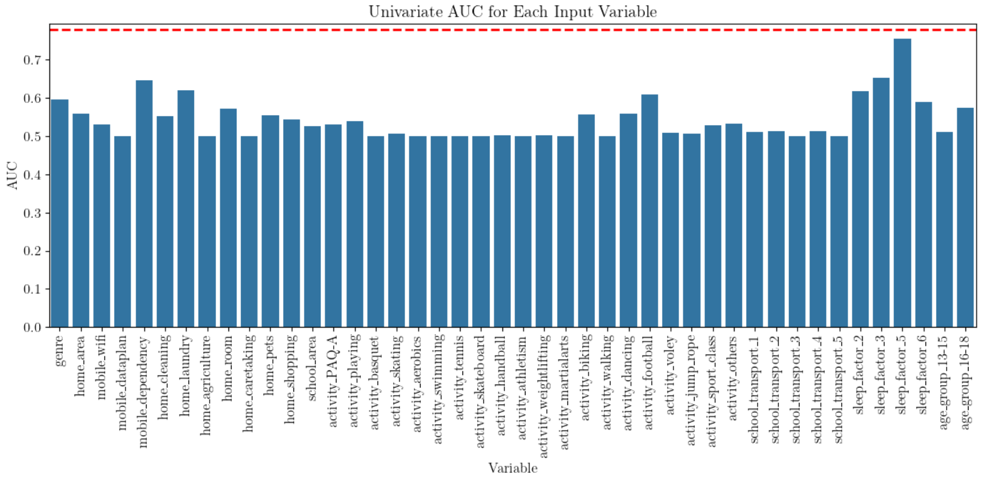
</td></tr>
<tr><td>Assess overfitting risk in the final model (e.g., via calibration plots or Brier score) and report sensitivity/specificity for each risk scale cutoff (Figure 4c).</td><td>We thank the reviewer for the comment. Indeed, we have strengthened our overfitting assessment by significantly changing our data usage during training and evaluation. In the new version of the manuscript, we have split our entire dataset into three subsets: a model training dataset (60%), used to perform a cross validation and train 60 different models (3-fold repeated 20 times) , the final model is an ensemble of these 60 models; a risk definition dataset (20%) used to define the risk thresholds based on the predictions of the model ensemble; and a risk evaluation dataset (20%) used to evaluate the effective prevalence of positive cases in each risk level. Our results show that the prevalence of positive cases in both risk definition and risk evaluation datasets is almost equal, which suggests that our risk model can be successfully extrapolated to other individuals. Notably, our model successfully splits the population into low risk (expected 2.3% prevalence), medium risk (expected 17.5% prevalence) and high risk (expected 47.8% prevalence) subgroups.</td></tr>
<tr><td>Test linearity assumptions for ORs (e.g., sleep_factor_5 OR = 7.89) and discuss coefficient instability indicated by wide CIs (e.g., [4.72–11.9])</td><td>As mentioned in previous replies, the OR have been corrected in the new version of the manuscript. Even though, the results regarding the most important variables in the risk determination model do not significantly change, the corrected ORs are much smaller than previously reported. The largest OR is obtained for the sleep_factor_3, which is 1.181 [1.086 – 1.302]. This value suggests that a person with the maximum score in this feature (3.2) is between 8.6% and 30.2% more likely to experience MSKP than a person with the minimum score in this feature (0). Given the much more conservative ORs, and owing to the fact that the results are an ensemble of 60 models, we are confident of our results and the stability of the model.</td></tr>
<tr><td>Use precise language (e.g., "associated with" instead of "correlated") to reflect logistic regression results accurately</td><td rowspan="2">We thank the reviewer for their comments. The entire discussion was reviewed, improving the writing style and addressing the four ideas suggested by the evaluator: balancing the emphasis on mobile dependence, explaining football as a protective factor, differentiating the proposed index from other tools, and providing a more critical engagement with prior studies.</td></tr>
<tr><td>Balance emphasis on mobile dependency (OR = 1.72) relative to stronger predictors like sleep factors, avoiding overhyping its role.</td></tr>
</table>

Hypothesize mechanisms for football's protective effect (OR = 0.66), such as aerobic fitness or coordination, to deepen interpretation.	
Engage critically with prior studies (e.g., explain differences from Thailand/Japan findings) rather than offering superficial comparisons.	
Benchmark the MSKP index against existing tools (e.g., Nordic Musculoskeletal Questionnaire) to substantiate novelty claims.	
Specify the MSKP index's intended application (e.g., "for screening high-risk adolescents") rather than using vague terms like "promising."	The conclusions section was reviewed and modified according to the suggestions.
Avoid causal language (e.g., "contributing to this risk") given the cross-sectional design, emphasizing the need for longitudinal validation.	
Acknowledge additional limitations: non-probabilistic sampling, lack of external validation, and potential overfitting risk.	The limitations sections was modified as follows: In future investigations, it would be advisable to increase the sample size by incorporating data from various schools located in both urban and rural settings to improve the representativeness and generalizability of the results. Additionally, although this study employed logistic regression models, there is potential to develop multiple regression models, such as multinomial logistic regression or more sophisticated predictive approaches, which can incorporate a broader range of associated variables and provide more accurate predictions of musculoskeletal risk. However, it is important to note that the risk factors and lifestyle habits evaluated in this study were self-reported by participants, which may introduce recall bias and subjective interpretations. Furthermore, the non-probabilistic sampling used, combined with the absence of external validation, may limit the external validity of the findings and increase the risk of overfitting of the model. To enhance the external validity and robustness of the MSKP index, future research should adopt a multicenter approach with strict eligibility criteria, thereby increasing accuracy and using cross-validation or external validation methods to reduce the risk of overfitting.
Clarify the ambiguous suggestion of "multiple regression models" (e.g., specify multinomial logistic regression or another approach).	
Complete funding details per PLOS ONE guidelines (e.g., include grant amount for Project ID: ENF-59-2020).	Since the process at this point is double-blinded, we have been required by the editors of the journal to remove the funding details.

Resolve the placeholder Harvard Dataverse DOI ("XXX") with a functional link.	The link to the data repository has now been updated to Uribe Laverde, Miguel Angel. 2025. “Muskuloesqueletal Risk Index.” Harvard Dataverse. https://doi.org/doi:10.7910/DVN/6NTV2M.
Update references to include key predictive modeling studies (e.g., Hestbaek et al., 2006; Dunn et al., 2013) and address timeline issues (e.g., remove or justify 2024 citations post-dating 2022 data collection)	Thank you very much for your valuable feedback. We have reviewed and improved the introduction regarding the knowledge gap, incorporating references from Hestbæk et al. and other recent authors to strengthen the theoretical foundation. Additionally, we included current bibliographic references, recognizing that although the fieldwork was conducted in 2022, the academic discussion on this phenomenon remains ongoing.

Reviewer #2:

Comment	Response
The study lacks a clear theoretical framework explaining how risk factors (e.g., mobile dependency, sleep disorders) contribute to MSKP. The global prevalence of adolescent MSKP should be briefly discussed to highlight the study’s significance.	The introduction now features a more robust problem statement, emphasizing that excessive screen exposure reduces physical activity and disrupts circadian rhythms, leading to poor sleep quality. This creates a vicious cycle: decreased rest results in higher daytime fatigue, which lowers energy and motivation to move, further promoting sedentary behaviours associated with screen use. This cycle not only contributes to the development of musculoskeletal pain but also impairs overall well-being and recovery. We are grateful for the additional important studies suggested by the reviewer and by yourself, as they will help improve the current version of the article and strengthen the scientific basis of our work.
The broad age range (10–18 years) is not justified. Adolescents experience different developmental changes, so age-stratified analysis is recommended.	We thank the reviewer for the suggestion. Indeed, as an early stage of our analysis we considered to separate the age groups and train a model for each group. However, we found that age-sensitive models do not perform better than a single model incorporating the age as a categorical variable. In addition, since the sample size gets even smaller, age-sensitive models exhibit much larger variability. The figures below show the obtained ROC curves for models trained either with individuals age 12 and younger or individuals age 13 and older. As can be seen, the performance of each model as measured by the AUC is worst than the model presented in the manuscript.

<table>
<tr>
<td></td>
<td>Model for 12 years old and younger:
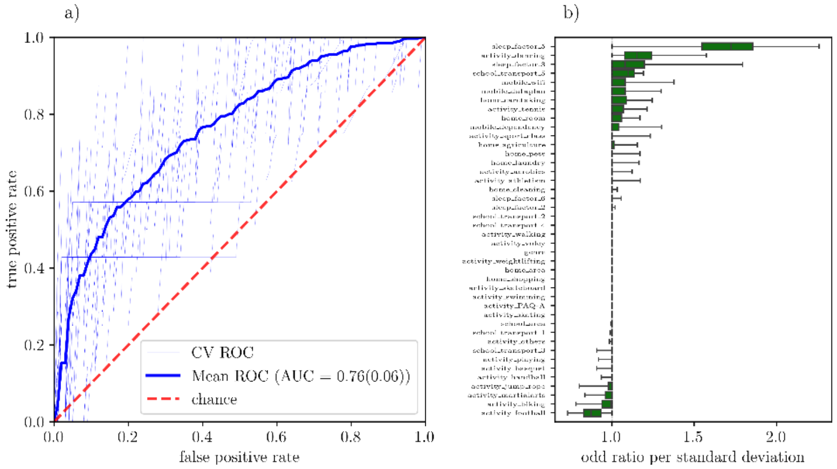

Model for 13 years old and older
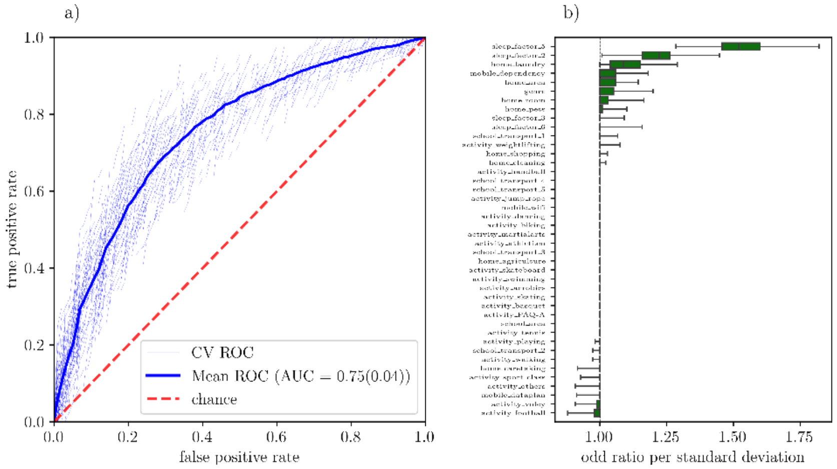

Since our goal is to propose the model with the largest possible discrimination power, we present in our manuscript the unified model where the age is introduced as an additional categorical variable.</td>
</tr>
<tr>
<td>The case definition (MSKP score ≥4) needs validation. Why was this threshold chosen?</td>
<td>Thank you for your valuable feedback. The threshold of MSKP score ≥4 was chosen based on preliminary analyses during the development phase of the index, which indicated that this cutoff best distinguished between low and higher-risk individuals in our sample. However, we acknowledge that this cut-off requires further validation through external datasets and longitudinal studies to confirm its predictive accuracy and generalizability. Future research will aim to validate this threshold across diverse populations and settings.</td>
</tr>
<tr>
<td>The feature selection method (Lasso Regression) should be justified against alternatives like PCA or stepwise regression.</td>
<td>We thank the reviewer for the comment. We want to emphasize that the goal of our research was to create a model with the largest power to discriminate the individuals experiencing pain to find the most relevant features related to pain occurrence. This means that we were looking for a model both accurate and interpretable. While PCA is a powerful tool to reduce dimensionality and</td>
</tr>
</table>

<table>
<tr><td></td><td>simplify the complexity of models, it affects the interpretability by creating new features that are a mixture of all original features.
On the other hand, when trying a stepwise regression, we obtain an over-simplified model (as compared to the Lasso regression) with only three relevant variables: sleep_factor_3, sleep_factor_5, and mobile_dependency. While these variables are the most relevant in our Lasso model, a model using only these three variables loses discrimination capacity (as suggested by the AUC curve on the risk evaluation set) and leads to an almost uniform score distribution for the positive cases, which results in weaker risk determination capabilities. We do not consider this experiment to contribute to our manuscript and we do not include it in our revised version.
The following figure shows the results of the stepwise regression model.
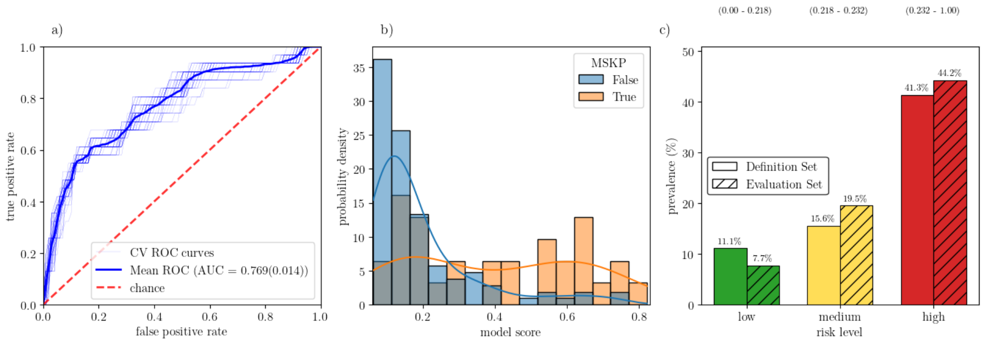
</td></tr>
<tr><td>Confounding factors (nutrition, mental health, socioeconomic status) are not considered but could influence MSKP risk.</td><td>The Limitations section has been extended to include confounding factors.</td></tr>
<tr><td>The 22.6% prevalence of MSKP should be reported with confidence intervals for accuracy.</td><td>We confirm that in our study, the prevalence of positive MSKP cases among the surveyed students was found to be 22.6%. This figure was derived from 154 students classified as positive cases of MSKP out of a total of 680 participants in the study. A positive case was defined as the presence of significant pain in two or more body locations.
To address your suggestion and quantify the uncertainty associated with this prevalence estimate, we have calculated the 95% Confidence Interval (95% CI) for this proportion.
We utilized the Wilson Score method to compute the confidence interval, as it is a robust and appropriate method for proportion estimates, especially given our sample size and observed proportion. This method provides more reliable limits compared to other approaches for proportions. Based on 154 positive MSKP cases out of a total of 680 students, the observed prevalence of 22.6% has a 95% Confidence Interval of [19.7% – 25.9%]. We have correspondingly included this CI in our manuscript.</td></tr>
<tr><td>Regression coefficients should include effect sizes and confidence intervals to assess the strength of associations.</td><td>We appreciate the comment. In the revised version of the manuscript, the reported regression coefficients are presented in all cases as a distribution obtained from the ensemble of cross-validation models. The Odd Ratios have also been calculated using these distributions and their respective confidence intervals have been Bonferroni corrected to consider the number of features</td></tr>
</table>

	included in the final analysis. From this analysis the impact of each variable, and its confidence interval, has been adequately assessed.
The logistic regression model (AUC = 0.83) is promising but needs calibration plots and sensitivity/specificity values to confirm robustness.	We thank the reviewer for the comment. To address the concern, we have adapted our data workflow to strengthen our analysis and confirm the robustness of the model. In the new version of the manuscript, we have split our entire dataset into three subsets: a model training dataset (60%), used to perform a cross validation and train 60 different models (3-fold repeated 20 times) , the final model is an ensemble of these 60 models; a risk definition dataset (20%) used to define the risk thresholds based on the predictions of the model ensemble; and a risk evaluation dataset (20%) used to evaluate the effective prevalence of positive cases in each risk level. Our results show that the prevalence of positive cases in both risk definition and risk evaluation datasets is almost equal, which suggests that our risk model can be successfully extrapolated to other individuals. Notably, our model successfully splits the population into low risk (expected 2.3% prevalence), medium risk (expected 17.5% prevalence) and high risk (expected 47.8% prevalence) subgroups.
The study suggests causal relationships, but given its cross-sectional design, only associations can be inferred.	We greatly appreciate the comment. The manuscript has been entirely revised to ensure that causal relationships are not suggested.
The sample is region-specific (Colombia), limiting generalizability. This should be acknowledged.	This limitation has also been included in the manuscript.
A stronger comparison with existing research would help contextualize the findings.	The comparison has been included in the manuscript.
The limitations section should be included, highlighting:<br>• The cross-sectional nature (no causal conclusions).<br>• The lack of objective measures for physical activity and sleep quality.	Both limitations have been included in the new version of the manuscript.
Should briefly mention key limitations (e.g., cross-sectional design, self-reported data).	Limitations have been included in the abstract.
Add scatterplots for key correlations (e.g., mobile dependency vs. MSKP risk).	We have included in the last figure the distribution of key features for negative and positive cases of MSKP to evidence the change in the distributions.
Ensure confidence intervals are included in tables.	All confidence intervals have been included in the final version of the manuscript.
Ensure citations follow PLOS One formatting and include key MSKP studies.	References have been updated

With best regards,

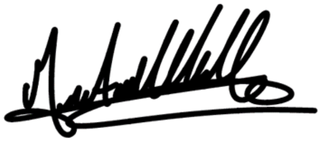

Miguel Angel Uribe Laverde
Associate Professor
Faculty of Engineering – Universidad de La Sabana
